# Stunting and Its Associated Factors among Children Below 5 Years Old on the East Coast of Peninsular Malaysia: Evidence from the National Health and Morbidity Survey

**DOI:** 10.21315/mjms2023.30.5.13

**Published:** 2023-10-30

**Authors:** Muhammad Zulfahmi Haron, Abdul Jalil Rohana, Noor Aman A. Hamid, Mohd Azahadi Omar, Noor Hashimah Abdullah

**Affiliations:** 1 Department of Community Medicine, School of Medical Sciences, Universiti Sains Malaysia, Kelantan, Malaysia; 2 Sector for Biostatistics and Data Repository Sector, National Institutes of Health (NIH), Ministry of Health Malaysia, Selangor, Malaysia; 3 Non-Communicable Disease Unit, Disease Control Branch, Kelantan State Health Department, Ministry of Health Malaysia, Kelantan, Malaysia

**Keywords:** children, factors associated, malnutrition, stunting

## Abstract

**Background:**

Child malnutrition problems still occur in Malaysia, particularly stunting. This study aimed to determine the proportion of stunting among children below 5 years old and investigate the factors associated with stunting on the East Coast of Peninsular Malaysia.

**Methods:**

This study utilised data from the 2016 National Health and Morbidity Survey (NHMS). Multiple logistic regression was used to determine the factors associated with malnutrition among non-stunted and stunted children.

**Results:**

The proportion of stunting among children below 5 years old in this East Coast region was 26.2%. When divided by state, Kelantan had the highest proportion of stunting, followed by Pahang and Terengganu, at 28.8%, 26.2% and 23.4%, respectively. In this study, the factors associated with stunting were children aged 24 months old–59 months old (adjusted odds ratio [aOR]: 1.52; 95% CI: 1.26, 1.83; *P* < 0.001), male children (aOR: 1.47; 95% CI: 1.23, 1.76; *P* < 0.001), Orang Asli children (aOR: 2.84; 95% CI: 1.86, 4.32; *P* < 0.001), children with low birth weight from 1,500 g to 2,499 g (aOR: 1.86; 95% CI: 1.36, 2.55; *P* < 0.001) and children from households that practice unsanitary waste disposal (aOR: 1.42; 95% CI: 1.16, 1.74; *P* = 0.001).

**Conclusion:**

Stunting among children under the age of 5 years old on the East Coast of Peninsular Malaysia remains a public health problem. To reduce the prevalence of stunting in this region, intervention programmes should be intensified. Emphasis should be placed on public health programmes that target the associated factors, such as dietary habits, Orang Asli children, low birth weight and unsanitary waste disposal.

## Introduction

Malnutrition accounts for nearly half of all global deaths among 5-year-old children (1). The global prevalence of stunting in children below 5 years old is 22.0% (2). In 2019, approximately 144 million children worldwide under the age of 5 years old are affected by stunting (1). Although the Malaysian national prevalence for stunting is 20.7%, three states on the East Coast region of Peninsular Malaysia recorded the highest prevalence: Kelantan (34.0%), Terengganu (26.1%) and Pahang (25.7%) (3). Furthermore, the prevalence showed an increase over 10 years, from 17.2% (2006) to 20.7% (2016), indicating that stunting is one of the major public health problems in Malaysia (3). In 2020, the Ministry of Health Malaysia released its National Health and Morbidity Survey (NHMS) report showing that the prevalence of stunting below 5 years old had increased to 21.8% (4).

According to the developmental origins of health and disease theory, children who experience stunting will have a higher likelihood of obesity and metabolic complications as they grow older due to decreased energy expenditure in their bodies (5). Those who survive have to deal with irreversible short- and long-term effects, including impairments in cognitive, motor and language development. Furthermore, their learning abilities and academic achievements will be affected. As these children become adults, their work productivity could negatively impact a country’s economy; they are prone to chronic illnesses that are costly to treat and increase mortality rates (6, 7).

Currently, little is known about stunting on the East Coast of Peninsular Malaysia. Although the prevalence of stunting in this region was above the national average, data are limited. Therefore, studies examining the determinants of stunting in this region are urgently needed. The objective of this study is to determine the proportion and factors associated with stunting among children below 5 years old on the East Coast of Peninsular Malaysia.

## Methods

### Data Source and Sampling

Study data were sourced from the primary data on Maternal and Child Health collected under the umbrella of the 2016 NHMS. In addition, with the criteria developed for this study, the data were re-analysed to investigate the factors associated with stunting among children below 5 years old on the East Coast of Peninsular Malaysia. The NHMS is a nationally representative health survey of the population that covers all districts in 16 states in Malaysia. It is a yearly population-based health survey designed to obtain information on the health status of Malaysians. The NHMS is routinely conducted by a team of researchers from the Institute of Public Health. The target population in the 2016 NHMS included all mothers aged 15 years old–49 years old with last childbirth less than 2 years before the survey and their children under 5 years old of age in the selected living quarters. The National Registration Department provided birth registration data from June 2014 to January 2015 to generate a sampling frame. The study participants included 2,829 mother-child pairs.

### Eligibility criteria

All children below 5 years old on the East Coast of Peninsular Malaysia who participated in the 2016 NHMS were included. However, respondents with more than 20% incomplete data were excluded.

### Data Collection and Variables Definition

Detailed data collection procedures have been described and published in the 2016 NHMS report (8). The data were collected by visiting households and conducting face-to-face interviews to obtain information on the participants’ demographic characteristics, child health and anthropometric data, household wealth, environmental factors and others (8).

Data related to the child (age, sex, ethnicity, birth weight and gestational age), mother (BMI, educational level and employment status), and household (strata, household income, main source of drinking water, types of toilets and types of waste disposal) were collected.

The anthropometric indicator height-for-age was used to determine nutritional status. Child stunting, normal and tall were defined by a height-for-age Z (HAZ) score: below −2 standard deviation (SD), above −2SD but below +2SD and above +2SD, respectively (9). In this study, the normal and tall parameters were categorised as the non-stunting group.

Variables were categorised by age in months (0–23 or 24–59), birth weight in grams (< 1,500; 1,500–2,499; 2,500–2,999; 3,000–3,999 and > 4,000) (10), gestational age (full-term or pre-term) and household income in RM (≤ 969.99; 970.00–1,999.99; 2,000.00–2,999.99; 3,000.00–3,999.99; 4,000.00–4,999.99, > 5,000.00) (11).

The participants were also categorised by ethnicity into three groups: i) Malay, the majority ethnicity in Malaysia; ii) Orang Asli, an indigenous group and iii) other ethnicities for Chinese, Indian, other Bumiputera and others based on the proportion of ethnic groups residing on the East Coast of Peninsular Malaysia.

Water sources were divided into ‘treated’ for piped water with a pipe into the house, piped water into a compound or yard, piped to a neighbour’s house, public standpipe, bottled drinking water (mineral or distilled), protected well and a tanker truck, and ‘untreated’ for unprotected well, rainwater collection, and river, stream, dam, canal, and irrigation channels (8).

Latrine types were grouped into ‘sanitary’ for flush toilets connected to the main sewerage system, flush toilets with a septic tank and pour flush toilets, and ‘unsanitary’ for pit or borehole with closed lid, pit or borehole latrine without cover or open pit, hanging toilet or toilet direct to sea or river and no toilet facility (8).

Waste disposal methods were categorised into ‘sanitary’ for waste collected by local authority or management either regularly or irregularly, waste buried outside the house, or waste collected and disposed into specialised areas for recycling, and ‘unsanitary’ for open burning, waste thrown into the drain, river, sea or anywhere else, and others (8).

### Statistical Analysis

Data were analysed using the IBM SPSS version 24.0 software. The children were divided into two groups, the non-stunting (which includes the normal and tall groups) and stunting groups. Numerical data were described using mean and SD, whereas categorical data were presented in frequency (*n*) and percentage (%). Median and interquartile range (IQR) were used for the numerical data, which were not normally distributed. To obtain the proportion of stunted children under 5 years old on the East Coast of Peninsular Malaysia, the following formula was used:


$$\text{Proportion of stunting}=\frac{\text{number of stunted children}}{\text{total sample size}}\times 100$$

To select the preliminary variables associated with the stunting group of children, a simple logistic regression was used. The non-stunting group (referent) was coded as ‘0,’ and the stunting group was coded as ‘1.’ For multiple logistic regression, variables with *P* < 0.250 from univariable analysis and clinically important variables were selected. The level of significance was set at *P* < 0.05.

After comparing the model using the above methods, the preliminary main effect model was obtained. The classification table and area under the receiver operating characteristic (ROC) curve were used to test the model fitness. A high overall percentage in the classification table and area under the curve approaching the value of 1 indicates that the model is fit (12).

The final model was determined by the forward and backward log-likelihood ratio method. Then, it was presented with an adjusted odds ratio and 95% confidence interval (CI), Wald statistics and *P*-value. The level of significance was set at *P <* 0.05.

## Results

Table 1 describes the characteristics of all participating children (*n* = 2,829). Of the children surveyed, 26.2% and 73.8% were stunted and non-stunted, respectively. The total distribution of children was divided almost equally among the East Coast states of Peninsular Malaysia: Kelantan (33.6%), Pahang (34.4%) and Terengganu (32.0%) (Table 1).

Most of the respondents were residing in rural areas, at 58.4% compared with those residing in urban areas at 41.6%. The mean age for the non-stunting group was 24.1 months (SD = 13.7), whereas that for the stunting group was 26.9 months (SD = 14.8). Sex was almost equally distributed in the non-stunting group, whereas the stunting group had more boys (58.5%) than girls (41.5%). Most respondents were Malays and fewer than 12.0% were non-Malays. Regardless of sex, most children had birth weights ranging from 3,000 g to 3,999 g and were born at full term.

Approximately half of the mothers in both groups had normal BMIs, but 41.0% were overweight and obese. In both groups, numerous parents had secondary education. Most mothers were unemployed in both groups, with 54.4% and 55.9% in the non-stunting and stunting group, respectively. The median household income for the non-stunting and stunting groups was RM2,500.00 (IQR = RM3,120.00) and RM2,300.00 (IQR = RM3,000.00), respectively. Both groups utilised treated water sources for daily usage (95.5%) and had sanitary latrines (97.6%) and sanitary waste disposal methods (68.9%).

The overall proportion of stunting among children below 5 years old on the East Coast of Peninsular Malaysia was 26.2%. When divided by state, Kelantan showed the highest proportion of stunted children in this region at 28.8%, followed by Pahang and Terengganu at 26.2% and 23.4%, respectively.

Table 2 demonstrates the results of the simple logistic regression examining the variables associated with stunting on the East Coast of Peninsular Malaysia among children under the age of 5 years old (Table 2). In the univariable analysis, strata, age, sex, ethnicity, birth weight, gestational age, father’s education, mother’s education, household income, water source, types of latrines and waste disposal types were found to be *P* < 0.250; these variables were included in the multiple logistic regression analysis.

The multiple logistic regression analysis showed that sex, age, ethnicity, birth weight and waste disposal methods were significantly associated with stunting among children below 5 years old on the East Coast of Peninsular Malaysia (Table 3). The odds of boys having stunting was 1.47 (95% CI: 1.23, 1.76; *P* < 0.001) compared with girls. In terms of age, the odds of stunting among children aged 24 months–59 months was 1.52 (95% CI: 1.26, 1.83; *P* < 0.001) compared with younger children in the 0 month–23 months age group. Compared with Malay children, the odds of being stunted among Orang Asli children was 2.84 (95% CI: 1.86, 4.32; *P* < 0.001) and children with other ethnicities were 42% less likely to have stunting (95% CI: 0.39, 0.87; *P =* 0.008). Compared with children with birth weights of 3,000 g–3,999 g, children with birth weights of 1,500 g–2,499 g had 1.86 (95% CI: 1.36, 2.55; *P* < 0.001) higher odds of being stunted. Households with unsanitary waste disposal methods had odds of being in the stunting group of 1.42 (95% CI: 1.16, 1.74; *P* = 0.001) compared with households with sanitary waste disposal methods. The classification table showed that the overall correctly classified percentage was 73.8%. The area under the ROC curve was 0.633 (95% CI: 0.610, 0.657). Thus, the model could accurately discriminate 63.3% of the cases.

## Discussion

The total proportion of stunting on the East Coast of Peninsular Malaysia among children under the age of 5 years old was 26.2%. When further divided into each state of the East Coast, the proportion of stunting was 28.8%, 26.2% and 23.4% for Kelantan, Pahang and Terengganu, respectively. These results revealed that the proportion of stunting among children in this vicinity (*n* = 2,829) is higher than the national and global prevalence (1). However, the proportion is slightly different compared with the previous NHMS 2016 results, indicating 34.0%, 26.1% and 25.7% for Kelantan, Terengganu and Pahang, respectively. The differences occurred due to the different denominator values when calculating proportion specifically for this study’s purposes. For the 2016 NHMS, the denominator utilised the entire population (*n* = 14,910) in Malaysia (3). In Myanmar, by using the 2015–2016 Demographic and Health Survey results, the stunting prevalence among children under the age of 5 years old was 29.1%. Divided by sub-national levels of 15 regions, the stunting prevalence ranged from 17.4% to 41.3% for each region (13).

The prevalence of stunting in Malaysia has shown an increasing trend over the past decade, that is, from 2011 (16.6%) to 2015 (17.7%) with a 6.6% rise, to 2016 (20.7%) with a 16.9% rise and the new prevalence was revealed in 2019 (21.8%) with a 5.3% increase (4, 14, 15). Nevertheless, this increase in prevalence could be attributed to a better sampling frame and larger sample size from year to year through the national survey. With this approach, more stunting cases could be captured to obtain a more accurate picture of the real issue of stunting in our children. A local study conducted by UNICEF Malaysia among children below 5 years old living in low-cost flats in urban Kuala Lumpur revealed that 22.0% of the children were stunted, with a higher prevalence noted among Chinese children (16). The higher proportion of stunting among Chinese children was possibly explained by the existing high-density population of Chinese people living in Kuala Lumpur (17). Without reinforcing and enhancing intervention or preventive programmes, this pattern is likely to grow. Consequently, reaching a 40% reduction globally in the stunting prevalence of children under 5 years old by 2025 will be impossible as illustrated in Global Nutrition Targets (18) and Sustainable Development Goals (1).

In this analysis, the proportion of stunting in children was higher in older children than in younger children. Because stunting is a sign of chronic undernutrition, it is common at an older age relative to other types of malnutrition. A recent study using 72 Demographic and Health Survey data involving 416,181 children in low- and middle-income countries found that stunting rates were higher among older children aged 6 months old–20 months old (50.7%) compared with younger children under 6 months old (17.8%) (19). However, one study indicated that stunting was prevalent in younger children. Compared with 24-month-old–59-month-old children, stunting was more likely to occur in children aged 11 months old–23 months old (20).

Another important finding from this study was sex; boys were at a higher risk of stunting than girls. It correlates with a systematic analysis by de Onis and Branca (6). Additionally, in Brunei, Boylan et al. revealed that male children have 2.36 greater odds of stunting than female children (21). Boys were more likely to have stunting due to gender differences in feeding pattern; particularly at age of 2 years old, boys consumed more than one serving of complementary daily feeding and potentially engaged more often in complementary daily feeding within a week than exclusive breastfeeding. Inadequate complementary feeding frequency leads to less nutrient intake than exclusive breastfeeding. This phenomenon can contribute to increased stunting among boys compared with girls (22). Moreover, the difference may be partly explained by the maternal-foetal environment and gender-specific development patterns. In the uterus, male foetuses devote a larger proportion of their resources to their development, putting them at an increased risk of malnutrition, low birth weight and probable linear growth failure (23). In contrast, a group of researchers observed that female children were more likely to be malnourished, particularly stunted than male children (24). A global review of diet indicators reveals inequalities in feeding and caretaking for girls compared with boys, leading to higher stunting prevalence among girls (25). This condition could be due to power relations and social norms perpetuating the discrimination of women.

Malaysia is a multiracial and multicultural country. These factors are essential for shaping nutritional status distribution, prevalence and determinants. Thus, the indigenous people Orang Asli were estimated to represent 13.8% of approximately 32 million Malaysians in 2015 (26). Inappropriate infant feeding practices and recurrent infectious diseases, particularly when surrounded by an unhealthy environment, could lead to nutritional deficiencies (27). For example, the prevalence of stunting among Orang Asli children in Gua Musang, Kelantan, was higher than among non-indigenous children (28). Another important factor related to stunting is food insecurity. Orang Asli children have several reasons to sustain traditional food intake; however, restricted forest access to traditional foods may lead to food insecurity issues (29).

This study found that infants with birth weights of 1,500 g–2,499 g have a 1.86 times higher risk of stunting. In a cross-sectional analysis using data from the Indonesian Family Life Survey on 4,101 children aged 2 years old–4.9 years old, researchers revealed that low birth weight was a contributing factor in stunting (30). In Myanmar, children with low birth weight have a 2.06 times probability to be stunted (13). Children with low birth weight are more likely to suffer from various infections, diseases, loss of appetite and nutritional deficiencies than children with normal birth weight. This condition could explain why children with low birth weight are vulnerable to stunting (31). Regarding the life course theory, any stimulation or change during embryo and foetal growth in the uterus can contribute to irreversible physiological, metabolic and structural changes, which may cause newborns to experience undernutrition and chronic diseases as they mature (32).

Other contributing factors to stunting include the use of untreated water and unsanitary latrines (33). In this study, children residing in households or areas with unsanitary waste disposal were at a 1.42 times greater risk of stunting compared with households with sanitary waste disposal methods. Unsanitary waste disposal methods may lead to recurrent fecal cross-contamination among households. This cross-contamination can lead to environmental enteropathy. This disease induces malnutrition and stunting by increasing bacterial permeability and decreasing nutrient absorption rates in the gastrointestinal tract system (34). A lack of safe drinking water, poor hygiene and impaired sanitation are significant risk factors for mortality (15). A systematic review on environmental factors for stunting showed that improper waste disposal is directly linked to stunting (35). However, in modern-day Malaysia, houses without a proper sanitary waste disposal system are rare. Houses without such amenities are more likely to be illegally constructed or constructed in a poor environment; thus, it is an indirect measure of poverty.

Although income is not a factor in this study, several related factors lead to stunting as proposed in the WHO Stunting Framework (7). Household income is one of the prominent determinants of stunting based on evidence from other countries (36). Globally, most studies found an association between stunting and poverty (37); however, other derived associated factors found in this study outweighed income, such as being an Orang Asli child and unsanitary waste disposal methods. Both of these factors acted as a proxy or an indicator of low household income and low education level (38). Another self-reported study also showed a similar finding: income is not associated with stunting (39). As shown in this NHMS study, the assessment of socioeconomic status by the self-report method as a subjective measure had a low concurrent validity (34). In a study investigating people from different socioeconomic levels and their propensity to report personal income, Turrell (40) found that the propensity not to report their income was greater among higher socioeconomic groups and lowest among the unemployed. Therefore, an objective measure of income is needed, rather than the traditional way of income-self-reporting basis in Malaysia. This measure can serve as a future direction in studying the association between stunting and income at the national level. The reason is that reporting non-objective incomes could have implications for health inequality research (40).

In Malaysia, stunting occurs in all socioeconomic classes, not only in poor families. For Malaysian households with income over RM5,000, the stunting prevalence is 17.4%, which was high relative to the upper-middle income countries’ level of 6.9% (14). In a cross-country analysis in Uganda, only the increase in self-employment income is linked to good nutritional outcomes relative to other sectors (36). In comparison, in India, nearly half of the children were stunted compared with only one-sixth in Senegal. India has been the world’s largest economic market with a gross national income (GNI) of USD3,620 per capita, whereas Senegal had a lower GNI. Senegal’s success in tackling malnutrition shows that political will, government assistance and partner support can decrease the number of children suffering from malnutrition, irrespective of economic status (41).

This study has its strengths. As previous data for stunting in the coastal part of Peninsular Malaysia was limited to small-scale studies, this study investigated child stunting on the East Coast of Peninsular Malaysia by using Malaysian national data. This study utilised broad population-based research and complex sample analysis, enabling results inferring the East Coast region. Although maternal BMI is one of the factors contributing to a child’s growth reported by previous studies (39), it is not significant in the current study. Due to restrictions on secondary data, other factors that lead to child malnutrition, such as diet quality, household dietary diversity, infant feeding and household food insecurity (6, 7) were not captured in this analysis. The limitations of the study include underlying problems of missing data and incomplete information, such as child morbidity and feeding practices, including exclusive breastfeeding. Furthermore, data on the number of households and the position of children in the family were not collected, which may also contribute to stunting. By redefining quintile household income as a selected collection of household assets, not only as direct cash to the household, and gathering data on household food insecurity, the nutritional status of children below 5 years old can be further evaluated. These factors may be included in future studies.

## Conclusion

The findings in this study based on national data provide the Ministry of Health and policymakers with evidence of the factors contributing to child stunting on the East Coast of Malaysia. Based on these results, initiatives should be centred on intervention programmes to improve nutrition among Orang Asli children, prioritise early detection, prevent low birth weight during pregnancy and improve existing local authority services for waste management. Nutrition-specific and nutrition-sensitive interventions are crucial to solving nutritional problems. Nutrition-sensitive interventions address the underlying causes of malnutrition, such as poverty and reducing food insecurity. Additionally, specific nutrition-related goals and actions should be initiated.

## Figures and Tables

**Table 1:** Characteristics of children below five on the east coast of Peninsular Malaysia (*n* = 2,829)

Variables	Total			Non-stunting (*n* = 2,087)			Stunting (*n* = 742)		
		
	Mean (SD)	*n*	%	Mean (SD)	*n*	%	Mean (SD)	*n*	%
State									
Kelantan		950	33.60		676	32.40		274	36.90
Pahang		974	34.40		718	34.40		256	34.50
Terengganu		905	32.00		693	33.20		212	28.60
Strata									
Urban		1,178	41.60		887	42.50		291	39.20
Rural		1,651	58.40		1,200	57.50		451	60.80
Age (month old)	24.88 (14.08)			24.12 (13.70)			26.93 (14.87)		
0–23		2,015	71.20		1,540	73.80		475	64.00
24–59		814	28.80		547	26.20		267	36.00
Sex									
Girls		1,364	48.20		1,056	50.60		308	41.50
Boys		1,465	51.80		1,031	49.40		434	58.50
Ethnicity									
Malay		2,420	88.70		1,801	89.40		619	86.70
*Orang Asli*		112	4.10		49	2.40		63	8.80
Other ethnicities^a^		197	7.20		165	8.20		32	4.50
Birthweight (gram)^b^	3.02 (0.47)			3.06 (0.46)			2.93 (0.48)		
< 1,500		26	0.90		15	0.70		11	1.50
1,500–2,499		282	10.00		173	8.30		109	14.70
2,500–2,999		914	32.30		664	31.80		250	33.70
3,000–3,999		1,550	54.80		1,186	56.90		364	49.10
> 4,000		55	1.90		47	2.30		8	1.00
Gestational age (week)	38.60 (1.51)			38.66 (1.40)			38.44 (1.74)		
Full term		2,651	93.70		1,974	94.60		677	91.20
Pre-term		178	6.30		113	5.40		65	8.80
Mother’s BMI	24.60 (5.24)			24.61 (5.15)			24.56 (5.48)		
Normal		1364	50.30		1,001	49.90		363	51.60
Underweight		235	8.70		170	8.40		65	9.20
Overweight and obese		1,113	41.00		837	41.70		276	39.20
Father’s education level									
No formal education/Primary		469	17.20		312	15.50		157	22.00
Secondary		1,618	59.40		1,219	60.60		399	55.90
Higher		639	23.40		481	23.90		158	22.10
Mother’s education level									
No formal education/Primary		380	13.50		238	11.40		142	19.10
Secondary		1,648	58.30		1,248	59.90		400	53.90
Higher		797	28.20		597	28.70		200	27.00
Mother’s employment status									
Employed		1,277	45.20		950	45.60		327	44.10
Unemployed		1,547	54.80		1,132	54.40		415	55.90
Monthly household income (RM)^c^	2,500.00 (3,200.00)^d^			2,500.00 (3,120.00)^d^			2,300.00 (3,000.00)^d^		
≤ 969.99		321	11.40		224	10.80		97	13.10
970.00–1,999.99		804	28.50		576	27.70		228	30.70
2,000.00–2,999.99		493	17.50		371	17.80		122	16.40
3,000.00–3,999.99		315	11.20		235	11.30		80	10.80
4,000.00–4,999.99		244	8.60		188	9.00		56	7.50
≥ 5,000.00		648	22.90		489	23.50		159	21.40
Source of water									
Treated water		2,702	95.50		2,013	96.50		689	92.90
Untreated water		127	4.50		74	3.50		53	7.10
Types of latrines									
Sanitary		2,760	97.60		2,048	98.10		712	96.00
Unsanitary		69	2.40		39	1.90		30	4.00
Types of waste disposal									
Sanitary		1,949	68.90		1,500	71.90		449	60.50
Unsanitary		880	31.10		587	28.10		293	39.50

**Table 2:** Simple logistic regression of factors associated with stunting among children below five years old on the east coast of Peninsular Malaysia (*n* = 2,829)

Variables	Regression coefficient (b)	Crude OR (95% CI)	Wald statistic	*P*-value
Strata				
Urban	0.00	1.00		
Rural	0.14	1.15 (0.97, 1.36)	2.43	**0.119**
Age (month old)				
0–23	0.00	1.00		
24–59	0.46	1.58 (1.32, 1.89)	25.30	**< 0.001**
Sex				
Female	0.00	1.00		
Male	0.37	1.44 (1.22, 1.71)	18.03	**< 0.001**
Ethnicity				
Malay	0.00	1.00		
Orang Asli	1.32	3.74 (2.55, 5.49)	45.27	**< 0.001**
Other ethnicities	−0.57	0.56 (0.38, 0.83)	8.29	**0.004**
Birthweight (gram)				
3,000–3,999	0.00	1.00		
< 1,500	0.87	2.39 (1.09, 5.25)	4.71	**0.03**
1,500–2,499	0.72	2.05 (1.57, 2.68)	27.90	**< 0.001**
2,500–2,999	0.20	1.23 (1.02, 1.48)	4.59	**0.032**
> 4,000	−0.59	0.56 (0.26, 1.18)	2.32	**0.128**
Gestational age				
Term	0.00	1.00		
Preterm	0.52	1.68 (1.22, 2.30)	10.20	**0.001**
Father’s education level				
Higher	0.00	1.00		
Secondary	0.00	1.00 (0.81, 1.23)	0.00	0.974
No formal education/Primary	0.43	1.53 (1.18, 1.99)	10.12	**0.001**
Mother’s education level				
Higher	0.00	1.00		
No formal education/Primary	−0.04	0.96 (0.79, 1.16)	0.20	0.658
Secondary	0.58	1.78 (1.37, 2.32)	18.59	**< 0.001**
Monthly household income (RM)^e^				
≥ 5,000.00	0.00	1.00		
4,000.00–4,999.99	−0.09	0.92 (0.65, 1.30)	0.24	0.622
3,000.00–3,999.99	0.05	1.05 (0.77, 1.43)	0.08	0.772
2,000.00–2,999.99	0.01	1.01 (0.77, 1.33)	0.00	0.935
970.00–1,999.99	0.20	1.22 (0.96, 1.54)	2.68	**0.102**
≤ 969.99	0.29	1.33 (0.99, 1.79)	3.55	**0.059**
Source of water				
Treated water	0.00	1.00		
Untreated water	0.74	2.09 (1.46, 3.01)	15.88	**< 0.001**
Types of latrines				
Sanitary	0.00	1.00		
Unsanitary	0.79	2.21 (1.36,3.59)	10.36	**0.001**
Types of waste disposal				
Sanitary	0.00	1.00		
Unsanitary	0.51	1.67 (1.40,1.99)	32.64	**< 0.001**

Note:

a based on National Poverty Line by Economic Planning Unit (EPU) (2016)

**Table 3:** Multiple logistic regression of factors associated with stunting among children below 5 years old on the East Coast of Peninsular Malaysia

Variables	Crude OR^a^ (95% CI)	Adjusted OR^b^ (95% CI)	Wald statistic^b^	*P*-value^b^
Age (month old)				
0–23	1.00	1.00		
24–59	1.58 (1.32, 1.89)	1.52 (1.26, 1.83)	19.15	**< 0.001**
Sex				
Female	1.00	1.00		
Male	1.44 (1.22, 1.71)	1.47 (1.23, 1.76)	17.95	**< 0.001**
Ethnicity				
Malay	1.00	1.00		
Orang Asli	3.74 (2.55, 5.49)	2.84 (1.86, 4.32)	23.51	**< 0.001**
Other ethnicities	0.56 (0.38, 0.83)	0.58 (0.39, 0.87)	7.10	**0.008**
Birthweight (gram)				
3,000–3,999	1.00	1.00		
< 1,500	2.39 (1.09, 5.25)	1.25 (0.52, 3.05)	0.25	0.619
1,500–2,499	2.05 (1.57, 2.68)	1.86 (1.36, 2.55)	15.21	**< 0.001**
2,500–2,999	1.23 (1.02, 1.48)	1.19 (0.98, 1.45)	2.99	0.084
> 4,000	0.56 (0.26, 1.18)	0.64 (0.30, 1.38)	1.29	0.257
Gestational age				
Term	1.00	1.00		
Preterm	1.68 (1.22, 2.30)	1.22 (0.83, 1.77)	1.02	0.312
Monthly household income (RM)^c^				
≥ 5,000.00	1.00	1.00		
4,000.00–4,999.99	0.92 (0.65, 1.30)	0.88 (0.61, 1.25)	0.53	0.466
3,000.00–3,999.99	1.05 (0.77, 1.43)	0.97 (0.70, 1.34)	0.03	0.863
2,000.00–2,999.99	1.01 (0.77, 1.33)	0.89 (0.67, 1.18)	0.66	0.417
970.00–1,999.99	1.22 (0.96, 1.54)	0.99 (0.77, 1.27)	0.01	0.924
≤ 969.99	1.33 (0.99, 1.79)	0.87 (0.62, 1.22)	0.68	0.409
Types of waste disposal				
Sanitary	1.00	1.00		
Unsanitary	1.67 (1.40, 1.99)	1.42 (1.16, 1.74)	11.60	**0.001**

Notes:

a Simple logistic regression;

b Multiple logistic regression;

c based on National Poverty Line by Economic Planning Unit (EPU) (2016)
